# Combined NGS and proteomics improves viral diagnostics in wildlife: coronaviruses confirmed in hedgehogs

**DOI:** 10.3389/fcimb.2026.1814090

**Published:** 2026-07-08

**Authors:** Ivan Chudinov, Elena Korneenko, Irina Rog, Alexandra Lukina-Gronskaya, Alexey Kovalenko, Veronika Gremyacheva, Ivan Butenko, Elena Vasileva, Daria Matyushkina, Elena Litvinova, Natalia Feoktistova, Anna Speranskaya

**Affiliations:** 1 Laboratory of Multiomics Research, Scientific Research Institute for Systems Biology and Medicine, Federal Service on Consumer Rights Protection and Human Well-Being Surveillance, Moscow, Russia; 2 Moscow Center for Advanced Studies, Moscow, Russia; 3 Laboratory of Simple Systems, Scientific Research Institute for Systems Biology and Medicine, Federal Service on Consumer Rights Protection and Human Well-Being Surveillance, Moscow, Russia; 4 Burnasyan Federal Medical Biophysical Center, Federal Biological Agency, Moscow, Russia; 5 Biological Department, Lomonosov Moscow State University, Moscow, Russia; 6 A.N. Severtsov Institute of Ecology and Evolution of the Russian Academy of Science, Moscow, Russia; 7 Laboratory of DNA Methylome and Transcriptome Editing, Vavilov Institute of General Genetics Russian Academy of Science, Moscow, Russia

**Keywords:** betacoronaviruses, *Coronaviridae*, hedgehogs, high-throughput sequencing, *Picornaviridae*, proteogenomics, proteomics

## Abstract

**Introduction:**

Metaviromic sequencing is prone to artifacts, necessitating orthogonal validation. This study evaluates a targeted proteogenomic approach for verifying viral presence initially detected by NGS.

**Methods:**

We analyzed oropharyngeal/anal swabs and feces from hedgehogs, where NGS indicated viruses from the *Coronaviridae*, *Picornaviridae*, *Flaviviridae*, and *Astroviridae* families. For verification, mass spectrometry was applied using sample-specific databases derived from NGS-assembled contigs.

**Results:**

Proteomic analysis confirmed *Coronaviridae* presence in NGS-positive animals, primarily detecting structural virion proteins in anal and fecal samples. However, proteomic evidence for *Picornaviridae*, *Flaviviridae*, and *Astroviridae* was not obtained despite their genomic detection.

**Discussion:**

Phylogenetic analysis of two complete *Picornaviridae* genomes assembled from the NGS data – one closely related to a UK rodent virus and another distantly related to Chinese bat viruses – supports the authenticity of the NGS findings. The results demonstrate that this two-stage pipeline provides critical orthogonal validation for metaviromic studies but also defines a practical sensitivity limit for the proteomic verification of some viral families. Combining NGS with proteomics significantly enhances the reliability of viral confirmation in complex samples, thereby strengthening ecological and zoonotic inferences.

## 1 Introduction

The advancement of high-throughput sequencing technologies has driven rapid growth in virological research, particularly in virome studies (Aulicino and Kimata, 2024). These studies are primarily exploratory, aimed at discovering novel viruses in diverse sample types or identifying and monitoring the spread of new genetic variants of known pathogens (Farkas et al., 2025; Kubacki et al., 2021; Tan et al., 2024). Methodologically, a significant portion of this research relies on reference-based assembly of raw reads or *de novo* contig assembly, followed by taxonomic assignment based on homology with known database entries (Wu et al., 2024). This approach generates crucial insights into viral distribution patterns, enabling the identification of natural reservoirs and the delineation of geographic areas with a higher probability of zoonotic disease emergence (Popova et al., 2025; Speranskaya et al., 2024; Ebinger et al., 2024; Parker et al., 2025).

However, conclusions drawn solely from NGS data require independent verification (Kubacki et al., 2021; Hirai et al., 2024; Zheng et al., 2021). Confirmatory evidence is especially critical in specific scenarios: the detection of closely related strains, sequences derived from low viral load, detection in an atypical reservoir, or cases of suspected contamination (Lukina-Gronskaya et al., 2024; van Zeggeren et al., 2021; Perlejewski et al., 2020; Thorburn et al., 2015; Sun et al., 2023; Jurasz et al., 2021).

Beyond these technical challenges, a more fundamental biological question often remains unanswered by nucleic acid tests alone: does the detection of viral RNA in swabs or feces signify an active, replicating infection within the host, or does it merely reflect passive environmental contamination or dietary intake? Many RNA viruses, particularly those with fecal-oral transmission routes, can pass through the gastrointestinal tract without establishing a productive infection. In such cases, the animal may act as a mechanical carrier, and the presence of viral nucleic acids could be transient, potentially leading to an overestimation of zoonotic risk. Therefore, differentiating between viral carriage and true viral replication is crucial for accurate epidemiological risk assessment.

The definitive verification of a novel virus traditionally requires its isolation in cell culture, followed by morphological and genetic characterization (Klyuchnikova et al., 2025; Mao et al., 2025). However, this approach is not universally feasible due to strict biosafety requirements, the absence of permissive cell lines for many viruses, and the frequent degradation of virion in field-collected samples (Hannemann, 2020; Tang et al., 2025). Consequently, there is a pressing need for robust, culture-independent methods to confirm viral discovery (Mark et al., 2024; Dsa et al., 2023).

For independent non-classic virology approach verification, methods based on the detection of molecules other than nucleic acids offer a valuable alternative. Among these, proteomic approaches that target stable viral protein markers have proven effective in virology, as established in foundational reviews (Maxwell and Frappier, 2007). The utility of this strategy was underscored during the COVID-19 pandemic, where mass spectrometry was successfully deployed to detect SARS-CoV-2 peptides in clinical swabs, serving as a complementary technique to PCR and ELISA (Pinto et al., 2021; McArdle et al., 2021; Ihling et al., 2020; Saadi et al., 2021).

A more integrated strategy, proteogenomics, directly uses proteomic data to validate and refine genomic inferences (Nesvizhskii, 2014). This approach has demonstrated significant value for tasks such as correcting genome annotations, characterizing virus-host interactions, and mapping the host response to infection (Xu et al., 2025; Bertzbach et al., 2019; Volkening et al., 2023; Pfaff et al., 2019). However, its widespread adoption remains constrained by a persistent methodological bottleneck: the difficulty of constructing high-quality, tailored sequence databases. Effective databases must be sufficiently comprehensive for accurate spectrum matching yet carefully curated to avoid excessive size, which compromises sensitivity and increases false-discovery rates (Jagtap et al., 2013; Castellana and Bafna, 2010).

This study aimed to develop and apply a shotgun bottom-up proteomics strategy to independently confirm the presence of viruses initially detected via total nucleic acid sequencing (DNA and RNA) of animal samples (swabs and feces). Our goal was to create a flexible, discovery-oriented method for the global identification of viral peptides within a sample, capable of confirming any virus identified through prior metavirome analysis. Unlike targeted screening assays, which require extensive development and validation of predefined peptide panels, this approach is inherently untargeted and adaptable. We adapted a proteogenomic pipeline previously validated for confirming plant-derived components in food products to the specific challenge of viral verification (Chudinov et al., 2026).

As key model targets, we focused on viruses from the families *Coronaviridae* and *Picornaviridae* that were identified by NGS-approach in a prior metavirome analysis of fecal and oropharyngeal swabs from hedgehogs, captured in European Russia (Lukina-Gronskaya et al., 2024). The rationale for this model system is twofold. First, it directly addresses a concrete need for verification, as the original NGS detection of these viruses, particularly under potential low viral load, requires orthogonal confirmation. Second, by applying a fundamentally different molecular principle — detection of viral peptides rather than nucleic acids — this work provides a higher level of confidence in the biological presence of these viruses within the host samples. Third, and most critically for veterinary epidemiology, proteomic detection enables us to probe the nature of the infection: the identification of viral structural proteins in excreta, particularly when coupled with the absence of certain non-structural proteins, can help distinguish between passive shedding and active viral replication. The detection of viral proteins could serve as indirect evidence of active viral replication and help clarify this question. Proteomic detection of viral proteins that are markers of active replication or persistent presence in tissues would provide strong independent evidence supporting this hypothesis.

The selected virus families are of significant public health interest due to their well-established zoonotic potential. Coronaviruses, particularly betacoronaviruses, are the causative agents of severe respiratory diseases in humans (e.g., MERS, SARS, SARS-CoV-2). Hedgehogs have been identified as carriers of betacoronaviruses across Europe, with EriCoV first described in Germany in 2012 and subsequently reported in France, Italy, the United Kingdom, Poland, Hungary and in Central part of European Russia (the easternmost finding of EriCoV in Europe to date) (Lukina-Gronskaya et al., 2024; Reuter et al., 2026). The most recent study further confirms the high prevalence (27.4%) of these viruses among hedgehog populations in Europe (Reuter et al., 2026). Moreover, a 2025 preprint provides evidence that the virus is capable of infecting hedgehog cells using the APN receptor, and demonstrates that certain strains (e.g., the Italian ErinCoV-12–19 and the British ErinCoV-UK-2014) actively replicated in pseudotyped cells expressing hedgehog APN (Jin et al., 2025). Thus, there is substantial reason to believe that the virus can replicate in wild hedgehog hosts. Their detection in hedgehogs, animals that often inhabit areas near human dwellings and are sometimes kept as pets, underscores the importance of monitoring these pathogens and validating the reliability of the methods used for their detection. However, as with any nucleic-acid based discovery, the fundamental biological question remains: does the presence of viral RNA signify active replication within the investigated wild animal.

Regarding the *Picornaviridae* family, its inclusion in the study was motivated by two main reasons: to test the universality of our method and the epidemiological context. Unlike enveloped (*Coronaviridae*), picornaviruses are non-enveloped and possess distinct physicochemical properties, including virion stability and capsid protein composition (Zell et al., 2017). Including this family allows us to test how universally applicable the proposed proteomic approach is for detecting viruses with fundamentally different structures, which is critical for assessing the method’s broad potential. Picornaviruses represent one of the most common and genetically diverse groups of viruses, infecting both humans (e.g., rhinoviruses, enteroviruses) and animals. Their frequent detection in metavirome studies, including in hedgehogs, makes them a convenient and relevant model for validating confirmation methods. Verifying their presence with proteomic methods is important for assessing the true scale of circulation of this heterogeneous group of viruses in wild animal populations.

In this study, we performed an *in silico* selection of viral peptides based on the obtained genomic data, followed by shotgun bottom-up mass spectrometry to detect these peptides. Here, we evaluated the feasibility of using targeted shotgun mass spectrometry as a “post-metaviromic” method to orthogonally validate NGS-based detections of novel viruses in complex animal samples. By comparing genomic and proteomic findings across different viral families, we aimed to establish not only a verification pipeline but also a framework for interpreting what the presence – or absence – of viral proteins truly means in the context of wildlife infection and carriage.

## 2 Materials and methods

### 2.1 Sample collection

The methods employed for the capture and swabbing of animals in this study has been approved by the Ethics Committee of the Research Institute for Systems Biology and Medicine (RISBM), protocol number N1.

A total of 16 hedgehogs (*Erinaceus* sp.) were included in this study. Animals were captured by professional zoologists during late spring and summer 2023 in various urban and suburban areas of European Russia. Capture was performed manually in the animals’ natural habitat during evening hours using handheld flashlights and protective gloves. Each animal was examined immediately at the capture site. The geographic locations of capture, sample IDs, and additional metadata are provided in **Supplementary Table 2**. After sampling, all hedgehogs were released unharmed back into their natural habitat. Oral (herein and later referred with prefix -o) and anal (-a) swabs were taken, after which they were immediately released into their natural habitat unharmed.

Two of the animals (namely, 23_5(MOS) and 23_23(MOS)) were kept in animal-care center, so swabs and feces were collected on the day of arrival.

Since all animals were captured in the Eastern European sympatry zone – where *Erinaceus europaeus* and *Erinaceus roumanicus* co-occur and hybridize – we refrained from assigning captured individuals to a specific species without dedicated genetic analysis. Therefore, throughout this study, we refer to the examined animals as *Erinaceus* sp.

### 2.2 RNA extraction

All 26 samples were processed individually, without sample pooling at any step from RNA extraction through library preparation. RNA extraction was performed as previously described (Lukina-Gronskaya et al., 2024). Briefly, viral RNA was extracted from individual samples using the QIAamp Viral RNA Mini Kit (Qiagen, Germany). The resulting RNA extracts were then treated with DNase I (RNase-free; New England Biolabs, USA) according to the manufacturer’s instructions to remove residual genomic DNA. A total of 10 *µ*L of the resulting nucleic acid mixture was used for the subsequent reverse transcription step.

### 2.3 Library preparation and high-throughput sequencing

Library preparation was performed for individual samples as previously described (Lukina-Gronskaya et al., 2024). Briefly, the first strand of complementary DNA (cDNA) was synthesized using REVERTA-L (Amplisens, Russia). Subsequent steps were performed using the NEBNext Ultra II Directional RNA Library Prep Kit for Illumina (New England Biolabs, USA) in conjunction with the MGIEasy DNA Adapters-96 (Plate) Kit V1.0 (MGI, China). Following adapter ligation, USER enzyme (New England Biolabs, USA) was added to each reaction. The final libraries were amplified using the PCR Enzyme Mix and PCR Primer Mix provided in the MGIeasy FS DNA Library Prep Kit (MGI, China).

Sequencing was performed on a DNBSEQ-G400 platform (MGI, China) using the DNBSEQ-G400RS High-Throughput Sequencing Set (FCL PE150). Raw FASTQ files available at SRA NCBI online server under BioProject accession PRJNA1309645. Complete genomes are available at NCBI GenBank under accessions PX448256, PX448257, PV359182-PV359184. See **Supplementary Table S2** for correspondence between accession numbers and sample names.

### 2.4 Bioinformatic analysis of sequencing data

Sequencing data were processed using the “VERA” pipeline as previously described (Chudinov and Speranskaya, 2025). Briefly, the pipeline performed initial read trimming followed by filtering to remove low-quality reads, host sequences (*Erinaceus europaeus*, accession number GC_950295315.1), and potential contaminants (SARS-CoV-2, accession number NC_045512.2). The resulting clean reads were taxonomically classified using Kraken2 (v2.1.3) (Wood et al., 2019) and assembled *de novo* with SPAdes (v4.0.0) (Prjibelski et al., 2020).

Assembled contigs were taxonomically classified using a two-step approach. First, an initial ORF-based classification was conducted with MMseqs2 (v15.6f452) against the UniRef90 database (Steinegger and Söding, 2017, 2018; Suzek et al., 2007). Contigs identified as potentially viral were subsequently subjected to a more stringent, whole-contig nucleotide-level classification using a custom MMseqs2 database built from the RefSeq Viral dataset (O’Leary et al., 2016, 2024).

The classification results were parsed using JupyterLab (v4.1.4) and SeqKit (v2.9.0) to identify and extract contigs belonging to the following viral families of interest: *Coronaviridae*, *Arenaviridae*, *Adenoviridae*, *Rhabdoviridae*, *Poxviridae*, *Filoviridae*, *Flaviviridae*, *Picornaviridae*, and *Herpesviridae* (Jupyter, 2016; Shen et al., 2016, 2024).

Contigs assigned to these viral families were analyzed for potential extension by mapping the clean reads back to the contigs using Bowtie2 (v2.4.4), and by mapping contigs to each other or to reference sequences using Minimap2 (v2.27-r1193) with the -ax asm20 preset (Langmead and Salzberg, 2012; Li, 2018). As the specific extension strategy was sample-dependent, a comprehensive description is provided in the **Supplementary Material**.

Kraken2 results were post-processed by first excluding viral families with fewer than 20 assigned reads in a given sample. Read counts for the remaining families were normalized to the total number of reads per sample and then scaled to reads per million (rpm) for standardized reporting (see **Supplementary Material** and **Supplementary Table 1**) (Lukina-Gronskaya et al., 2024).

### 2.5 Generation of an assembly-based databases and inclusion lists

To generate databases and peptide inclusion lists for proteomic analysis, sample-specific amino acid sequence databases were constructed using a custom Python script. For each animal, the database comprised two components: (1) target sequences, consisting of predicted open reading frames (ORFs) and proteins from assembled viral contigs and genomes of interest, and (2) background sequences, including the host proteome (*Erinaceus europaeus*, accession number GCF_950295315.1) and proteins from non-target contigs. Contigs comprising complete or partial viral genomes were omitted from this process, as they were included at the first import of sequences. These animal-specific databases were used for subsequent mass spectrometry data identification.

Using BioPython (v1.85), pandas (v2.3.1), and pyteomics (v4.7.5), all protein sequences were subjected to an *in silico* tryptic digestion, allowing no missed cleavages and retaining peptides between 5 and 50 amino acids in length (Cock et al., 2009; McKinney et al., 2010; Reback et al., 2020; Goloborodko et al., 2013; Levitsky et al., 2018). To isolate peptides unique to the target viruses, any viral peptide also present in the background proteome was removed. This filtering was facilitated by representing the peptide sequences as a presence-absence matrix using CountVectorizer from scikit-learn (v1.5.1) (Pedregosa et al., 2011). Example of peptide generation notebook is available at GitHub. The final set of target-unique peptides was used to create inclusion lists for targeted HPLC-MS/MS analysis.

### 2.6 Protein extraction and precipitation

Proteins were extracted from animal swab and fecal samples by bead-beating homogenization in lysis buffer, consisting of 1X PBS, 5% SDS, 10 mM EDTA, 0.1 mM PMSF. Samples were adjusted to a volume of 250 *µ*L with Milli-Q water as needed and transferred to 2.0 mL Bead-Beating DuraTubes™ (SSIbio, USA) containing eight ceramic beads of 2 mm in diameter. Subsequently, 500 *µ*L of lysis buffer was added to each tube. Homogenization was performed using a Minilys homogenizer (Bertin Technologies, France) with three cycles of 1 minute at maximum speed, interspersed with 5-minute cooling intervals at 4°C. The homogenate was centrifuged at 10000 × *g* for 10 minutes at room temperature to pellet insoluble debris and reduce SDS foam. A 200 *µ*L aliquot of the resulting supernatant was collected for protein precipitation.

Proteins were precipitated using a TCA-acetone method. The precipitation solution (PS) consisted of 100% (*w/v*) TCA in acetone with 0.07% (*v/v*) 2-mercaptoethanol, and the rinsing solution (RS) was 0.07% (*v/v*) 2-mercaptoethanol in acetone. For precipitation, the 200 *µ*L of supernatant aliquot was combined with 200 *µ*L of ice-cold PS and 600 *µ*L of ice-cold RS in a 1.7 mL microcentrifuge tube. The mixture was vortexed vigorously, briefly centrifuged to collect the contents, and incubated overnight at −20°C to precipitate the proteins. The precipitate was pelleted by centrifugation at 10000 × *g* for 10 minutes at 4°C, and the supernatant was discarded. The pellet was washed by gently flicking the tube with 1 mL of RS (vortexing was avoided to prevent pellet disintegration). This centrifugation and washing step was repeated twice for a total of three washes. Finally, the protein pellet was air-dried for 10 minutes at room temperature prior to further processing.

### 2.7 Protein digestion

Following TCA-acetone precipitation, proteins were denatured for 30 minutes in a buffer containing 8 M Urea, 2 M Thiourea, 10 mM Tris (pH = 8). Protein concentration was estimated using a Bradford assay, and the entire precipitated protein amount from each sample was used for subsequent analysis (see **Supplementary Table 4**). Disulfide bonds were reduced by incubation with 15 mM DTT at 56°C for 30 minutes. Subsequently, cysteine residues were alkylated with 30 mM iodoacetamide at room temperature for 30 minutes in the dark. The reduction step was then repeated by adding a fresh aliquot of DTT and incubating at room temperature.

Following alkylation, samples were diluted fivefold with 50 mM NH_4_HCO_3_ (pH = 8) and digested with trypsin (Gold, Mass Spectrometry Grade, Promega, USA) at an enzyme-to-protein ratio of 1: 50 (*w/w*) overnight at 37°C. Protease activity was quenched by acidification with trifluoroacetic acid (TFA) to a pH ∼ 2. The resulting peptide mixtures were desalted using Copure C18A SPE Cartridge $100\;\frac{mg}{mL}$ microcolumns (Biocomma, China) and dried under vacuum.

### 2.8 HPLC-MS/MS analysis

Peptide analysis was performed using an Orbitrap Exploris 480 mass spectrometer (Thermo Fisher Scientific, USA) coupled online to an Ultimate 3000 RSLCnano chromatographic system (Thermo Fisher Scientific, USA) via a Nanospray Flex ion source (Thermo Fisher Scientific, USA). An initial data dependent acquisition (DDA) analysis was performed for all samples in one replicate. Following a six-month storage period at +4°C, a secondary targeted analysis using sample-specific inclusion lists.

Dried peptide samples were re-dissolved in 35 *µ*L of loading solvent (5% acetonitrile, 0.1% formic acid). Chromatographic separation was achieved using a reversed-phase setup comprising a C18 PepMap 100 precolumn (5 mm × 300 *µ*m, 5 *µ*m particles, 100Å; Thermo Fisher Scientific, USA) and a Peaky-C18 capillary analytical column (50 cm × 75 *µ*m, 1.9 *µ*m particles; Molekta, Russia). For each sample, a 5 *µ*L aliquot was loaded onto the precolumn at a flow rate of $10\;\frac{\mu L}{min}$ for 12 minutes using the loading solvent. Peptides were then eluted onto the analytical column at a flow rate of $250\;\frac{nL}{min}$ with a linear gradient of solvent B (80% acetonitrile, 0.1% formic acid) against solvent A (5% acetonitrile, 0.1% formic acid) as follows: 5 − 10% B over 6 min, 10 − 55% B over 108 min, and 55 − 65% B over 6 min. The column was subsequently washed with 100% B for 2 min and re-equilibrated with 5% B for 15 min.

The mass spectrometer was operated in a data-dependent acquisition (DDA) mode with a 1-second cycle time. Full-scan MS spectra ( $\frac{m}{z}\;200-1\;500$) were acquired at a resolution of 60000. Ions were selected for fragmentation based on a pre-calculated inclusion list of target peptides. This list was generated using the R package Peptides (v2.4.6) (Osorio et al., 2015) and contained monoisotopic masses of 2+ and 3+ charged ions, accounting for potential tryptic peptides with zero, one, or two missed cleavages and carbamidomethylated cysteine modifications. Precursor ions matching a target mass within a ±10 ppm tolerance were prioritized for fragmentation. If no target ions were present, the most abundant ions were selected instead. Dynamic exclusion was enabled to prevent repeated sequencing of the same precursor.

MS/MS spectra were acquired at a resolution of 15000 using a normalized collision energy of 30% and an isolation window of $1.4\;\frac{m}{z}$. The ion source voltage was set to 2200 V, and the ion transfer tube was maintained at 275°C. Automatic gain control (AGC) was set to “Standard” and maximum injection time to “Auto” for both MS and MS/MS levels. The Funnel RF Level was set to 50. The mass spectrometry proteomics data have been deposited to the ProteomeXchange Consortium via the PRIDE partner repository with the dataset identifier PXD067983 (Perez-Riverol et al., 2025).

### 2.9 Mass spectrometry data analysis

Tandem mass spectra were analyzed using the FragPipe computational platform (v22.0) in DDA mode (Kong et al., 2017; Teo et al., 2020). Spectra from both standard and inclusion list-based acquisitions were searched for each animal group against their corresponding, sample-specific custom database (described in the Section 2.5). All databases were supplemented with common contaminants and reversed protein sequences to enable false discovery rate (FDR) estimation.

Search parameters were configured as follows. Tryptic digestion was specified, allowing up to two missed cleavages. Peptide length was restricted to 5–50 amino acids. Precursor and fragment mass tolerances were set to 20 ppm and 5 ppm, respectively, with an isotope error tolerance of 0*/*1*/*2. Fixed modifications included carbamidomethylation of cysteine (+57.02146 Da). Variable modifications included oxidation of methionine (+15.9949 Da, max 3 occurrences), N-terminal acetylation (+42.0106 Da, max 1 occurrence), phosphorylation (+79.96633 Da, max 3 occurrences), pyro-Glu from Q or loss of ammonia at peptide N-terminal (−17.0265 Da, max 1 occurrence), pyro-Glu from E (−18.0106 Da, max 1 occurrence), and deamidation (+0.984016 Da, max 1 occurrences). Max variable modification combinations – 5000, max variable modifications per peptide – 4.

Peptide-spectrum matches (PSMs) were validated using Percolator, and protein inference was performed with ProteinProphet (Käll et al., 2007; Nesvizhskii et al., 2003). The results were filtered to a 5% false discovery rate (FDR) at the PSM, peptide, and protein levels (da Veiga Leprevost et al., 2020). Label-free quantification (LFQ) was performed using IonQuant, with the match-between-runs feature disabled (Yu et al., 2021).

### 2.10 Visualization

All the figures were generated using Python and the following packages: numpy (v1.26.3), pandas (v2.3.1), matplotlib (v3.8.2), seaborn (v0.13.2) (Van Rossum and Drake, 2009; Harris et al., 2020; McKinney et al., 2010; Reback et al., 2020; Hunter, 2007; Waskom, 2021). Genomic data visualizations were created with pyBedTools (v0.10.0) and the Integrative Genomics Viewer (IGV, v2.19.4) (Dale, 2024; Robinson et al., 2011; Thorvaldsdóttir et al., 2013). Final figure composition and editing were performed in Inkscape (Inkscape, 2025).

## 3 Results

### 3.1 Sequencing and assembly

We analyzed 26 samples (feces and swabs) from 16 animals of the genus *Erinaceus* (see Section 2.1 for sample descriptions). Viral load was assessed via total nucleic acid extraction, high-throughput sequencing, and a subsequent bioinformatic pipeline as detailed in the Materials and Methods section. This pipeline was designed for the taxonomic classification of reads and the identification of fragments or complete genomes belonging to various viral families.

**Figure 1:**
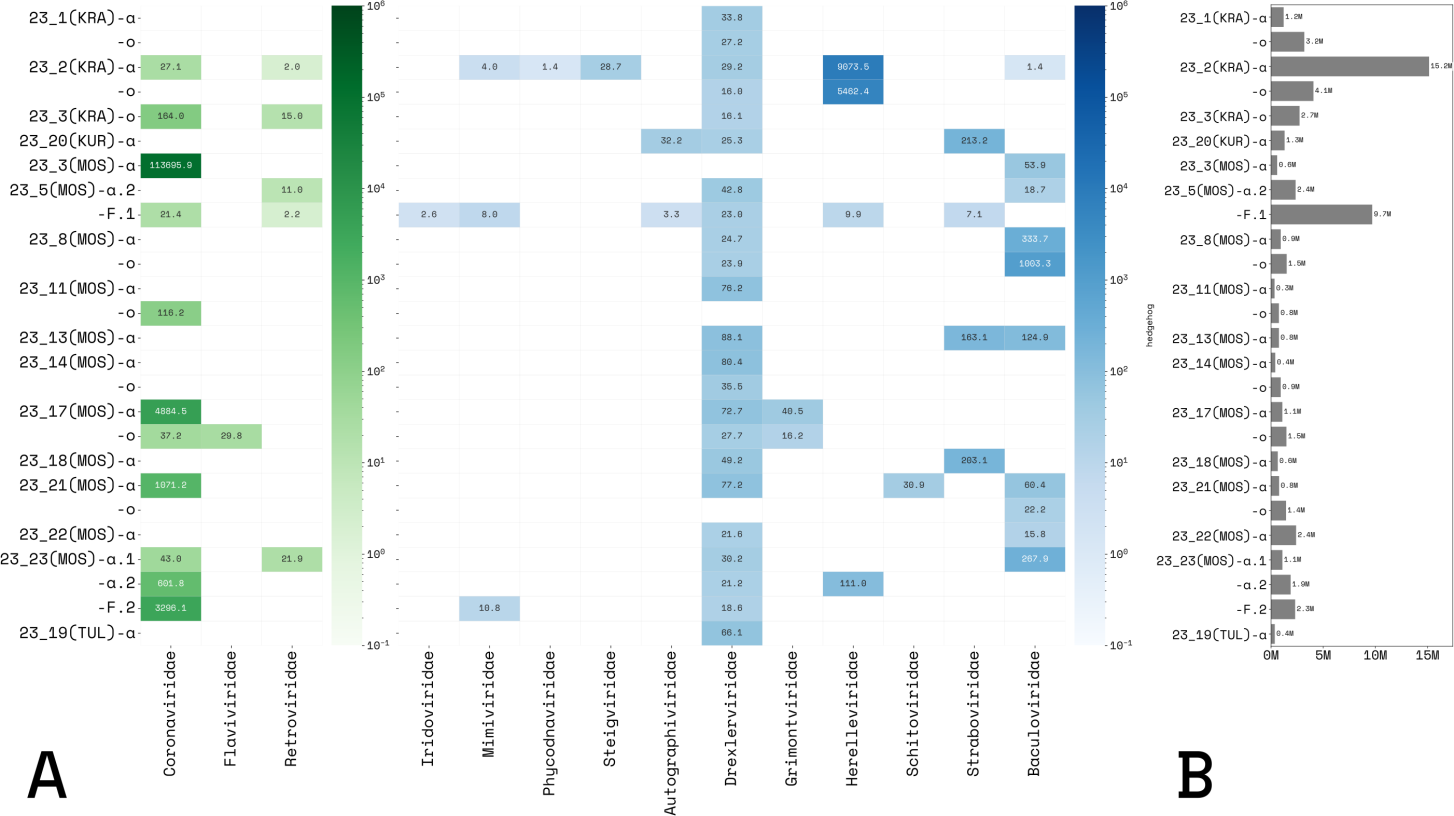
Metaviral sequencing overview. Relationship between total read count and relative read abundance assigned to a particular viral family. **(A)** Read-based taxonomic profiling of metaviromes using Kraken2 (threshold – 20 reads) from oral and anal swabs analyzed by high-throughput total RNA sequencing. The abundance of viruses in individuals is expressed as the number of Kraken2 classified reads per million total reads (rpm). Each column represents a virus family, while each row represents an individual animal caught in the region (in brackets) for oral (prefix -o) and anal (prefix -a) swabs. Repeating sample names are omitted for convenience. Columns with viral families of special interest are separated and shown in green (left), while other viral families are shown in blue (right). **(B)** barplot representing number of host- and contaminant- cleaned, quality filtered reads for each sample.

Metavirome analysis revealed a characteristically low viral load for the target zoonotic families in most samples. The majority of viral reads across all samples corresponded to bacteriophages. We observed that the successful assembly of complete genomes for the target families showed a weak relationship with the total number of non-host (or “clean”) reads (see **Figure 1**, **Supplementary Table 1**).

For instance, in sample 23_2(KRA)-a, which had the highest total read count (64.5 M), clean reads comprised 15.2 M (∼ 23%). Of these, only 27.12 rpm (411 reads) were classified as *Coronaviridae*, resulting in a failed coronavirus genome assembly. In contrast, sample 23_3(MOS)-a, with a moderate total read count (3.1 M) and a small proportion of clean reads (0.6 M), yielded a complete coronavirus genome (30 057 nt) because the vast majority of its clean reads belonged to *Coronaviridae*.

We found that a combined approach — initial viral load estimation with Kraken2 followed by dual taxonomic contig filtering — was effective for selecting the most promising samples for in-depth analysis. Ultimately, this analysis identified three complete *Coronaviridae* genomes (in samples 23_3(MOS)-a, 23_17(MOS)-a, and 23_23(MOS)-F.2) and two complete *Picornaviridae* genomes (in sample 23_5(MOS)-F.1). For other samples, only partial genome fragments could be assembled for these families, as well as for *Flaviviridae* (sample 23_17(MOS)). One sample (23_18(MOS)-a) contained a fragment belonging to the *Astroviridae* family. Accession numbers for the deposited genomes, along with the sequences of short contigs not deposited in NCBI but used for peptide generation, are provided in **Supplementary Table 2** and **Supplementary File**.

### 3.2 Peptide selection

To independently verify the findings from NGS, we developed an approach for selecting strictly specific viral peptides (see Section 2.5 and **Supplementary Material**). This process excluded peptides present in the host (*Erinaceus europaeus*) proteome or within predicted ORFs from other contigs in the same sample. For each sample, two databases were constructed: a target database (viral proteins) and a background database (host proteins and all predicted ORFs). For instance, for the coronavirus from sample 23_3(MOS)-a, 587 unique peptides and 32 non-unique peptides were obtained. Peptides such as FNVAVTR were marked as non-unique due to their presence in host proteins. Each protein of this virus was represented by at least three unique peptides. For samples with fragmented genomes, target peptides were generated based on closely related complete genomes, accounting for identified non-synonymous substitutions. Consequently, a final set of 92 to 903 unique peptides was defined for each sample group (see **Supplementary Table 3**), which was used to generate inclusion lists for targeted mass spectrometry.

### 3.3 Proteomics

For proteomic analysis, we selected 9 samples from 5 animals representing distinct scenarios: (i) samples with complete coronavirus genomes, (ii) samples with fragmented genomes from coronaviruses and other viruses, and (iii) samples where NGS indicated viral presence but complete genome assembly failed. The transport medium was used as a control.

**Figure 2:**
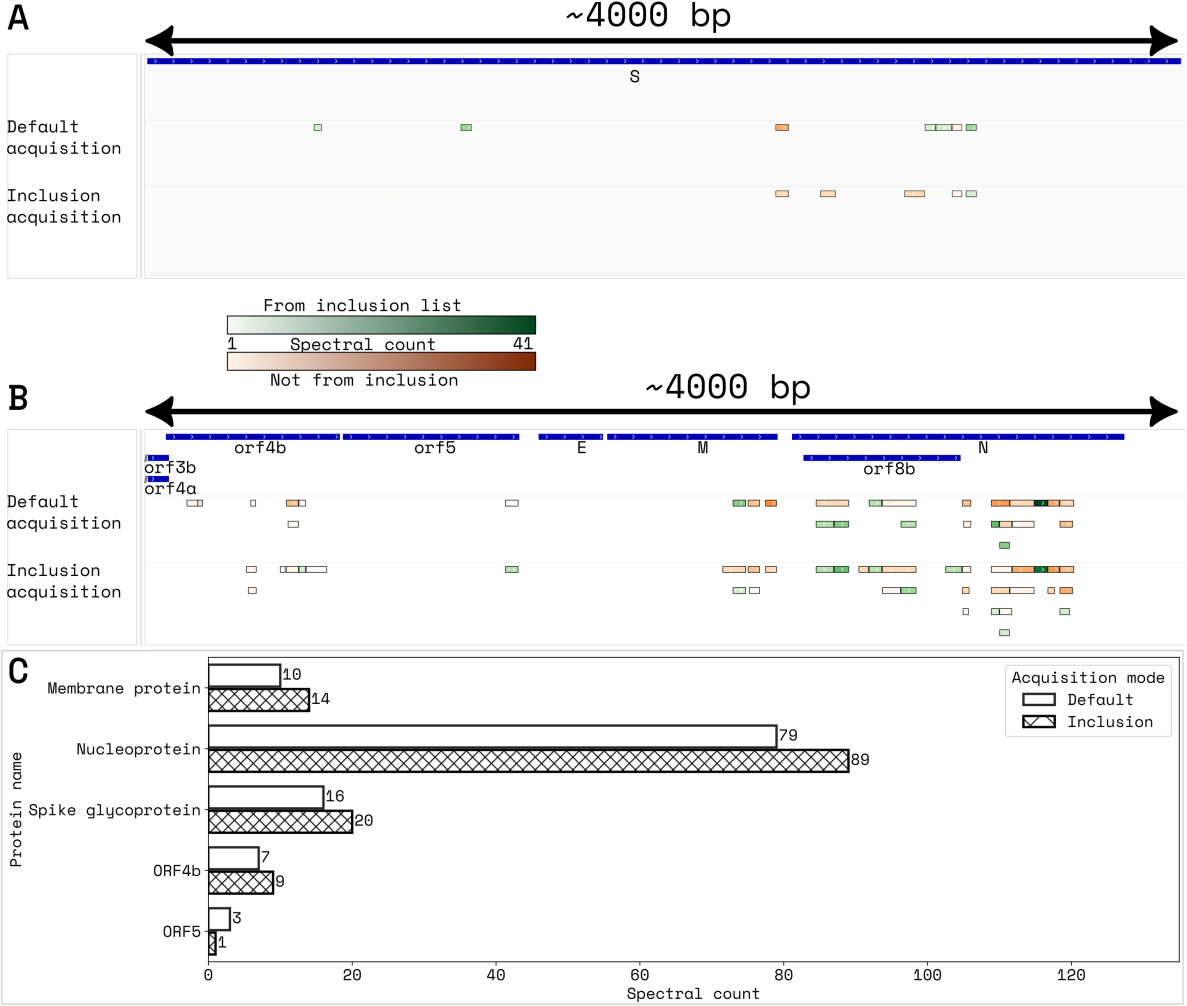
Representation of identified coronavirus peptides in 23_3(MOS)-a sample. **(A)** peptides supporting Spike-protein detection. **(B)** peptides supporting other structural proteins (E, M, N), ORF4b, orf5 and orf8b. In both **(A, B)** proteins from annotation are shown in blue bars with white arrows indicating ORF direction (as in IGV). Peptides are shown as horizontal bars at the corresponding protein positions, green bars represent peptides from inclusion list, orange bars represent other detected peptides (missed cleavages). Horizontal arrows show scale in base pairs. **(C)** Barplot of spectral counts for peptides from 23_3(MOS)-a coronavirus proteins. Blank patches represent default spectra acquisition, hatched patches correspond to inclusion list acquisition.

The majority of peptides identified in all samples matched proteins of the European hedgehog (*Erinaceus europaeus*), based on searches against the specialized sample-specific databases. Among the target families, peptides from *Coronaviridae* family were successfully detected in all three animals (23_3(MOS), 23_17(MOS), 23_23(MOS)) that yielded complete genomes from NGS analysis. In sample 23_3(MOS)-a, peptides corresponding to 5 distinct viral proteins were identified, primarily the nucleoprotein and spike protein (see **Figure 2**). For animal 23_17(MOS), peptides from three coronavirus structural proteins were detected exclusively in the anal (asinus) swab, but not in the oral (oris) swab. In animal 23_23(MOS), peptides were present in only one of two oropharyngeal swabs (uniquely mapping to the nucleoprotein) and in the fecal sample, which contained peptides from the membrane and spike glycoproteins but not the nucleoprotein. Notably, peptides from the replicase polyprotein 1a/1ab were not detected in any sample.

For the other target virus families, the proteomic analysis did not confirm the genomic findings. Despite the NGS-based detection of complete (*Picornaviridae* in 23_5(MOS)) or fragmented (*Flaviviridae* in 23_17(MOS), *Astroviridae* in 23_18(MOS)) genomes, corresponding viral peptides were not identified in any sample.

In summary, proteomic confirmation of *Coronaviridae* presence was achieved in 3 of 4 NGS-positive animals. Detection was heterogeneous across sample types from these animals, with peptides confirmed in 1 of 2 samples from animal 23_17(MOS) and in 2 of 3 samples from animal 23_23(MOS). No viral peptides were detected in samples containing only fragmented *Coronaviridae* genomes (23_5(MOS)). Overall, the proteomic method confirmed viral presence in 4 out of the 8 samples that were positive by NGS for *Coronaviridae*, while yielding no confirmation for *Picornaviridae*, *Flaviviridae*, or *Astroviridae* (see **Table 1**).

**Table 1:** Pivot summary of proteomics applications for metaviral research.

		*Coronaviridae*		*Picornaviridae*		*Flaviviridae*		*Astroviridae*	
Animal	Samples information	NGS	MS	NGS	MS	NGS	MS	NGS	MS
23_3(MOS)	23_3(MOS)-a	Complete	Detected	–	–	–	–	–	–
23_5(MOS)	23_5(MOS)-a.2	–	Not det.	Frag.	Not det.	–	–	–	–
	23_5(MOS)-F.1	Frag.	Not det.	2 compl.	Not det.	–	–	–	–
23_17(MOS)	23_17(MOS)-a	Complete	Detected	–	–	Frag.	Not det.	–	–
	23_17(MOS)-o	Frag.	Not det.	–	–	Frag.	Not det.	–	–
23_18(MOS)	23_18(MOS)-a	–	–	–	–	–	–	Frag.	Not det.
23_23(MOS)	23_23(MOS)-a.1	Frag.	Not det.	Frag.	Not det.	–	–	–	–
	23_23(MOS)-a.2	Frag.	Detected	–	Not det.	–	–	–	–
	23_23(MOS)-F.2	Complete	Detected	–	Not det.	–	–	–	–

In the proteomics (MS) section, blue cells indicate detection of corresponding peptides, while red cells indicate no detection. Cells with a dash (–) signify that the viral family was not within the analytical scope for that sample. Green cells in the genomics (NGS) section highlight complete genomes; yellow cells represent genome fragments. In the proteomics (MS) section, blue cells indicate detection of corresponding peptides, while red cells indicate no detection. Cells with a dash (–) signify that the viral family was not within the analytical scope for that sample.

## 4 Discussion

The primary aim of this study was to test an idea that a targeted, hypothesis-driven proteomic approach informed by primary NGS analysis can serve as an effective method for independent verification of virus detection, thereby substantially increasing the reliability of metaviromic research. To test this, we performed a hybrid analysis: from 26 samples collected from 16 animals of the genus *Erinaceus* sp., for proteomnic analysis we selected 9 samples from 5 animals that revealed NGS-detected virus families of significant public health interest due to their well-established zoonotic potential. No physical filtration (e.g., 0.45 *µm*) was performed prior to nucleic acid extraction. This was a deliberate choice, as our objective was to capture not only free virions but also viruses potentially residing within host cells or tissues. Physical filtration would have removed these cell associated particles. Instead, contamination control was implemented through a combination of bioinformatics filtering and orthogonal validation. Bioinformatics pipeline included: host read removal, protein based filtering, nucleotide based classification of remaining contigs against the viral database and manual curation of all candidate viral contigs. Furthermore, as the primary scope of this study, we performed orthogonal proteomic validation using HPLC-MS/MS on selected samples to obtain peptide level confirmation of viral presence.

Our data further illustrate a well-known challenge in metaviromic studies: high variability in data output despite the application of standardized protocols across samples (Buigues et al., 2024; Wang et al., 2023). This variability can be partly attributed to the complexity of multi-step sample preparation, where each stage can introduce bias into the sequencing results. Such multi-step processing is inevitable when the target of interest represents a minor component of the sample. Furthermore, inherent risks include laboratory contamination and high-throughput sequencing artifacts, such as computational cross-contamination of reads (Asplund et al., 2019; Holmes, 2019; Saremi et al., 2021; Haagmans et al., 2025; Konanov et al., 2025). Consequently, we assume that errors in metagenomic (metaviromic) sequencing represent a fundamental and unavoidable characteristic of such experiments. This underscores the necessity of confirming key findings using a strategy that validates NGS data with methods based on different physical principles than nucleic acid sequencing. The central objective of this work was to provide such independent verification of NGS findings using mass spectrometry, a high-throughput method for analyzing the protein component of a sample.

The proteogenomic approach successfully confirmed the presence of viruses from the *Coronaviridae* family in all three cases where NGS yielded complete genomes (animals 23_3(MOS), 23_17(MOS), 23_23(MOS)). In these samples, only peptides corresponding to structural virion proteins (e.g., nucleoprotein, spike, and membrane protein) were identified; peptides from non-structural replicative complex proteins (e.g., RdRp) were not detected. This pattern is likely attributable to the absence of replicative proteins within virions and, consequently, lower abundance in the sample Sender et al. (2021). The exclusive detection of structural proteins might suggest the absence of active viral replication in the host organism at the time of sampling. However, recent experimental studies using pseudovirus particles and screening of mammalian receptor orthologs in pseudotyped cell lines have demonstrated that EriCoV can enter hedgehog cells via the APN (aminopeptidase N) receptor, providing rather an evidence for active viral replication rather than passive carriage in these animals (Jin et al., 2025). Therefore, the identification of coronavirus structural proteins in our samples most likely supports this idea, indicating that these hedgehogs were indeed supporting viral replication and shedding infectious virions at the time of sampling.

Conversely, for other target families – *Picornaviridae*, *Flaviviridae*, and *Astroviridae* – proteomic confirmation was not obtained despite the detection of corresponding genomic contigs. For picorna- and flaviviruses, which have been previously described in hedgehogs, the most straightforward explanation is that the viral protein abundance in our samples was too low, insufficient for protein detection (Reuter et al., 2018; Hu et al., 2023; Schönbächler et al., 2019). This interpretation must consider a methodological nuance: for these viruses, peptide generation was based on the full annotated polyprotein without subdivision into self-cleaved subunits, which may have influenced the selection of optimal targets for detection and spectra identification. This factor, combined with inherent variability in peptide ionization or chromatographic behavior, makes it impossible to definitively distinguish between biological absence and technical difficulties for the mass spectrometry assay. The lack of proteomic confirmation for astroviruses, of which only a genomic fragment was detected, is consistent with data indicating no prior identification of this virus group in hedgehogs (Kuczera et al., 2024).

For *Coronaviridae*, based on our viral load estimation, samples below 600 rpm did not yield detectable peptides. In contrast, correspondng peptides were detected in all samples exceeding this threshold, suggesting a potential empirical lower bound for successful proteomic detection under our experimental conditions. However, any quantitative threshold must be interpreted with caution, as rpm reflects relative abundance rather than absolute viral copy number. Quantitative PCR remains the gold standard for targeted virus identification due to its high sensitivity and specificity, relatively low cost, and scalability, with limits of detection (LOD) around 100 copies*/*reaction for SARS-CoV-2 and as low as ∼ 4 copies*/*reaction for capripox viruses [Leonardi-Cattolica et al. (2024); Wen et al. (2023)]. In contrast, NGS enables untargeted virus discovery but faces sensitivity limitations and higher costs for routine surveillance, achieving LODs of ∼ 550 copies*/*mL in some applications [Tan et al. (2024)]. Recent advances in proteomics approaches reported LOD as low as limits of infectivity (∼ Ct 30) [Grossegesse et al. (2025)]. Successful peptide detection depends on numerous factors beyond viral load, including sample origin (e.g., swab type, fecal matrix), protein extraction and digestion efficiency, HPLC-MS/MS instrumentation and settings, fragmentation parameters, and the physicochemical properties of individual peptides. Additionally, the dynamic range of protein abundances within a sample profoundly affects detection probability [Abdul-Khalek et al. (2023); Schilling et al. (2026); Birhanu (2023)]. Together, these results highlight the importance of future comparative sensitivity assessment between methods and the need to define detection thresholds for combined NGS-proteomic approaches for different viral taxa, as has been done for SARS-CoV-2 (Arnaout et al., 2021).

The case of picornaviruses detected in animals 23_5(MOS) (two complete genomes in a fecal sample) should be discussed particularly. The presence of two complete genomes in sample 23_5(MOS)-F.1, with lengths of 8697 bp and 7655 bp (which falls within the typical range for the family according to ICTV), indicates a substantial amount of viral RNA in the sample (Zell et al., 2017). Comparative genomic analysis supports the validity of these findings: the first genome (23_5_MOS-F1_EriPic_A) showed 95.7% identity (with 100% coverage) to an unclassified picornavirus described in wild rodents captured in 2017 in the United Kingdom (accession number ON136177.1) (Raghwani et al., 2023). The high degree of similarity between viruses found in such geographically, temporaly and taxonomically distant hosts argues for the authenticity of our genomic discovery. The second genome (23_5_MOS_F1_EriPic_B) showing only distant similarity (∼ 30% identity over 71−73% of its length) to contigs assembled from metagenomic data of bats captured in 2017–2020 in China (accession numbers PP745899, PP745762), potentially indicating the discovery of a novel virus, which evolutionarily related to bat-borne picornaviruses (Wang et al., 2024). Thus, the totality of genomic data (completeness of assembly, phylogenetic context) provides compelling evidence for the actual presence of these picornaviruses in the samples. The subsequent lack of proteomic confirmation, despite seemingly favorable conditions for detection (high RNA content) does not refute the genomic finding but demonstrates the existence of a practical sensitivity limit for the proposed proteogenomic approach and underscores the importance of a independent assessment of proteomic detection threshold for different viral families.

While the application of inclusion lists did not yield a significant increase in the number of peptide identifications, likely due to sample aging, the approach remains promising. The aging is likely attributable to peptide degradation or adsorption to tube walls during the six-month interval between the primary and re-analysis (with inclusion lists), compounded by limited original sample volumes that precluded a complete new preparation. Nonetheless, the results demonstrate that application of inclusion lists for re-analyzing samples stored at 4°C in a water-acetonitrile mixture remains viable and provides comparable data. It can be hypothesized that employing inclusion lists during the analysis of freshly prepared samples would increase proteomic coverage, indicating the promise of this methodology when applied in a timely manner. Such experiments remain a focus for future investigation.

Various computational strategies exist for proteomic detection of viruses. Task-appropriate database construction is one of the central challenges in proteomic analysis. Different strategies have been proposed, including multi-stage spectrum identification, iterative identification, the use of *de novo* sequencing, database generation based on metagenome assembly graphs, and iterative spectral identification with automated genomic data integration (Jagtap et al., 2013; Zhang et al., 2016; Potgieter et al., 2023; Chiang and Collins, 2024; Tang et al., 2016; Saadi et al., 2021). Each of these approaches is optimized for a specific tasks. One previously proposed strategy include the workflow to select specific peptides for subsequent identification in a sample (Lechner et al., 2019). However, the method proposes selecting unique peptides using pre-built, universal “target” and “background” databases that contain all possible homologous peptides from closely related viruses. In contrast, we generate our set of unique peptides from the genomic sequences that distinguish the specific organism of interest, as identified by NGS sequencing of the very same sample subjected to proteomic analysis. The key distinction of our approach is its NGS-hypothesis verification-centric design. Unlike methods aimed at the *de novo* discovery or broad viral screening, we start with a specific genomic signal (from NGS of the same sample) and generate a tailored database to confirm the presence of that specific agent. This shifts the goal from ‘what viruses are here?’ to ‘is this particular NGS-identified virus truly present?’, addressing a critical need in metaviromic research. This approach involves the detection by untargeted mass spectrometry of all possible peptides theoretically predicted from that same genomic assembly. Thus, our work suggests a novel application of the proteogenomic paradigm specifically for the task of independently validating metaviromic results. We argue that the proposed approach is an effective tool for increasing the reliability of metaviromic studies, which could consequently reduce the number of reports of pathogen findings in unexpected reservoirs that later fail to be confirmed.

We believe the demonstrated method can significantly enhance confidence in virus detection across various ecological niches, thereby improving conclusions about the epidemiology of zoonotic disease spread. Furthermore, we hope this approach can refine understanding of zoonotic disease host models, helping to answer the question of whether an animal is a passive carrier (e.g., through the food chain) or is experiencing an active, albeit asymptomatic, infection. Nevertheless, while the sample set in the present study was sufficient to test the feasibility of our proteogenomic verification pipeline, it limits the generalizability of our conclusions regarding method performance across all wildlife viromics contexts. Proteomics was intentionally applied only to samples with prior NGS evidence of target viruses, as our aim was orthogonal confirmation rather than discovery. Consequently, future studies involving larger and more diverse sample collections will be necessary to establish the sensitivity and specificity of this approach on a broader scale.

In summary, the two-stage genomic-proteomic approach presented in this work is an effective tool to improve the reliability of metaviromic research. It not only confirms viral presence but also, by analyzing the profile of detected proteins, aids in more accurately differentiating between passive carriage and active infection in host animals. However, as noted above, the current methodology provides confirmation of viral presence but cannot definitively prove the absence of viral proteins, as this would require additional validation procedures. Despite this inherent limitation, the strategy significantly expands the researcher’s capabilities and supports more robust conclusions regarding the epidemiology and risk assessment of emerging zoonotic diseases.

## Data Availability

The datasets presented in this study can be found in online repositories. The names of the repository/repositories and accession number(s) can be found in the article/**Supplementary Material**.
