# Supplementary material for: Combined NGS and proteomics improves viral diagnostics in wildlife: coronaviruses confirmed in hedgehogs
Source: Front Cell Infect Microbiol. 2026 Jul 8;16:1814090. doi: 10.3389/fcimb.2026.1814090 (PMC13388059; doi:10.3389/fcimb.2026.1814090)
Supplement: Supplementary file 1 [file Presentation1.pdf]

Supplementary Material

1 READ-BASED TAXONOMIC PROFILING OF HEDGEHOG METAVIROMES

Taxonomic assignment was performed with Kraken2 on sequencing reads following the removal of host-derived and contaminant sequences. The corresponding data is available in Supplementary Table S1.

- **List A.** Raw read counts assigned to each viral family prior to the application of abundance thresholds.
- **List B.** Summary statistics of the cleaned read sets: total number of reads, cumulative sum of read lengths, and average read length.
- **List C.** Abundance of viral families of special interest, expressed in parts per million reads (ppm) after applying abundance thresholds.
- **List D.** Abundance of all other detected viral families, expressed in parts per million reads (ppm) after applying abundance thresholds.

2 DATA AVAILABILITY AND SUPPLEMENTARY FILE INFORMATION

See the Supplementary Table S2 to access the accession numbres.

3 ASSEMBLY-BASED PEPTIDES GENERATION RESULTS

See the Supplementary Table S3 to access the peptide generation results.

4 PROTEIN CONCENTRATIONS ESTIMATED USING A BRADFORD ASSAY

See the Supplementary Table S4 to access the protein concentrations.

5 REVIEW OF BIOINFORMATICS ANALYSIS RESULTS

5.1 Sample 23_3(MOS)-a

De novo assembly yielded three contigs of lengths 28631, 1196, and 395 bp. These contigs were analyzed using the online BLASTn tool against the NR database (Camacho et al., 2009; Benson et al., 2012), identifying the closest reference sequence as Betacoronavirus Erinaceus isolate EriCoV/RU/MOW15-2/2022 (accession number PP421221.1). The contigs were mapped to this reference using minimap2 (v2.27-r1193) with the `-ax asm20` preset (Li, 2018), revealing that they were overlapping and constituted a complete genome. To validate the assembly, the cleaned reads were mapped back to the assembled genome using Bowtie2 (v2.4.4) (Langmead and Salzberg, 2012). Genome annotation was performed with VAPiD (v1.6.7) and manually curated (Shean et al., 2019). The complete genome sequence was deposited in GenBank (accession number PX580474, Supplementary Table S2).

5.2 Samples 23_5(MOS)

This animal was represented by two samples: an asinus swab from day two (23_5(MOS)-a.2) and a feces sample from day one (23_5(MOS)-F.1). No *Coronaviridae* contigs were identified in the asinus swab. However, 34 *Coronaviridae* contigs were assembled from the fecal sample. Although subjected

to the same analysis pipeline as sample 23_3(MOS)-a, these contigs provided insufficient coverage to assemble a complete or draft genome. As the primary aim of these actions was to construct a database for proteomics, the clean reads from this sample were mapped to the complete 23_3(MOS)-a EriCoV genome. Variant calling was performed using BCFtools (v1.19), identifying three single-nucleotide polymorphisms (SNPs) passing filters (`INFO/DP > 5 & QUAL > 20`): one synonymous mutation in *orf1ab*, one missense mutation in the nucleoprotein (S410L), and one non-coding mutation near the genome end. All proteins predicted from the 23_3(MOS)-a EriCoV genome annotation were included in the custom database, with the nucleoprotein point mutation introduced. These partial genome sequences are available as Supplementary Data but were not deposited in GenBank (Supplementary Table S2 and Supplementary File).

Additionally, *Picornaviridae* contigs were identified: six contigs ($500 - 8753$ nt) in the fecal sample and three contigs ($265 - 343$ nt) in the asinus swab. As these contigs showed no identity to each other and *Picornaviridae* genomes typically range from 7.1 to 8.9 kb, complete genomes were not recreated from contigs (Zell et al., 2017; De Castro et al., 2024). Contigs longer than 5000 nucleotides were annotated using ORF homology; shorter contigs were used only as templates for ORF prediction. The complete *Picornaviridae* sequences were deposited in GenBank (accession numbers PX448256 and PX448257); fragments are available as Supplementary Data (Supplementary Table S2 and Supplementary File).

5.3 Samples 23_17(MOS)

Oris and asinus swabs from the same day were analyzed. A complete *Coronaviridae* genome (30251 bp) was assembled from the asinus sample (23_17(MOS)-a), alongside 14 *Coronaviridae* fragments ($212 - 521$ bp) from the oris sample (23_17(MOS)-o). Clean reads and contigs were mapped to the novel 23_17(MOS)-a EriCoV genome as described above. Several minor SNPs were identified in the oris contigs relative to the reference genome; however, these positions had low sequencing depth ($1 - 3$X) and were not consistent, so they were excluded from further analysis. The complete genome was annotated with VAPiD and manually curated, and the predicted proteins were used for downstream analysis. The complete genome sequence was deposited in GenBank (accession PX580475, Supplementary Table S2).

Flaviviridae contigs were also identified: five contigs ($404 - 1942$ bp) in the oris sample and twelve contigs ($232 - 816$ bp) in the asinus sample. Local BLASTn analysis revealed no identities between contigs. Given the typical *Flaviviridae* genome size of $9.7 - 12$ kb, a complete genome was not assembled, and the contigs were used as templates for ORF prediction (De Castro et al., 2024). These fragments are available as Supplementary Data and were not deposited in GenBank (Supplementary Table S2 and Supplementary File).

5.4 Sample 23_18(MOS)

Initial analysis identified a contig classified as *Poxviridae*, which was excluded upon manual curation. Subsequently, a contig of 4933 bp classified as *Astroviridae* was detected. Although this family was not initially a target, the contig was annotated using Prokka (v1.14.6), and the predicted proteins were included in the proteomics database (Seemann, 2014). This partial sequence was not deposited in GenBank and is available as Supplementary Data (Supplementary Table S2 and Supplementary File).

5.5 Samples 23_23(MOS)

Two consecutive daily asinus swabs (a.1, a.2) and one fecal sample from the second day (F.2) were analyzed. A complete *Coronaviridae* genome was assembled from the fecal sample (23_23(MOS)-F.2), while several fragments were identified in the asinus swabs (12 and 21 contigs in a.1 and a.2, respectively).

Contigs and clean reads were mapped to the complete genome as described previously. Minor deviations were observed in the contigs from sample a.2, but these were poorly supported by read depth coverage and were therefore disregarded. The complete genome was annotated with VAPiD and manually curated, and the predicted proteins were added to the database. The complete genome was deposited in GenBank (accession number PX580476, Supplementary Table S2).

Two short *Picornaviridae* contigs (233 and 237 bp) were identified in sample a.1. These were deemed too short for formal annotation and were used only for ORF prediction and subsequent database construction. These fragments are available as Supplementary Data and were not deposited in GenBank (Supplementary Table S2 and Supplementary File).

6 PEPTIDE SPECIFICITY

In addition to the standard proteomic analysis for validating sequencing results from animal swab and fecal samples, we conducted a comprehensive peptide specificity analysis. Given the expected dominance of host background proteins and bacterial metagenomic components, we performed a comparative assessment of peptide uniqueness. This involved *in silico* trypsin digestion of viral proteins and comparison against the complete host proteome and predicted ORFs from metagenomic contigs.

For each sample, we generated sets of unique peptides for use in inclusion lists, enabling selective precursor ion targeting during spectral acquisition (Hodge et al., 2013; Jaffe et al., 2008). For animals with multiple samples (differing by swab type or collection time), we performed a pooled contig and genome analysis. This approach, based on the assumption of persistent viral infection, aimed to identify peptides unique to viruses within that specific animal. To achieve this, we constructed a sparse matrix mapping viral peptides against the complete peptide set derived from all contigs detected in that animal.

6.1 Exemplary Workflow: Sample 23_3(MOS)-a

To illustrate, consider sample 23_3(MOS)-a — the only sample from animal 23_3(MOS) from which a complete *Coronaviridae* genome was assembled. All predicted proteins from this virus were included in the database for mass spectrometry data identification. To evaluate peptide uniqueness, we created two sequence groups:

1. Target proteins: All annotated proteins of the coronavirus from sample 23_3(MOS)-a.
2. Background database: All available proteins of the European hedgehog (*Erinaceus europaeus*) and ORFs from contigs obtained for this animal (excluding contigs incorporated into complete genomes), irrespective of their taxonomic origin.

This approach reliably distinguished virus-specific peptides from host or other source peptides. For example, the peptide `FNVAVTR`, while present in the coronavirus Replicase polyprotein 1ab, is also found in *Erinaceus europaeus* helicases. Therefore, its detection cannot serve as unambiguous proof of viral presence. Consequently, for the coronavirus in sample 23_3(MOS)-a, we identified 587 unique peptides and 32 non-unique peptides. Each protein of the novel coronavirus was represented by at least three unique peptides, confirming identification specificity (see Figure S1 and Supplementary Table S3).

6.2 Methodological Adaptations for Other Samples

The described method, with adaptations, was applied to other samples.

For sample 23_5(MOS), which contained numerous disjointed *Coronaviridae* contigs, the target peptide database was created based on coronavirus proteins from sample 23_3(MOS)-a. Virtual amino acid substitutions corresponding to non-synonymous mutations identified in the NGS data were incorporated. The background database for this animal was formed from all contigs derived from samples 23_5(MOS)-a.2 and 23_5(MOS)-F.1.

For samples 23_17(MOS), 23_18(MOS), and 23_23(MOS), the approach was modified. In addition to the full ORF set from all animal swabs, the analysis included ORFs predicted for contigs assigned to the *Flaviviridae*, *Astroviridae*, and *Picornaviridae* families, respectively. This allowed us to account for viral sequences for which complete genomes – and thus full annotation—could not be obtained. As a result, a set of unique peptides was defined for each sample group analyzed (Supplementary Table S3).

Based on this analysis, inclusion lists of target peptides were generated for each sample. These lists are intended for subsequent use in bottom-up mass spectrometry with instrument-based target ion enrichment, enabling confirmation of viral protein presence in the samples.

7 CORONAVIRUS DETECTION IN SAMPLE 23_23(MOS)

Proteomics confirmed the presence of coronavirus peptides (spike protein, membrane protein, and nucleoprotein), while picornavirus peptides were not identified (see Figure S2). It is noteworthy that the distribution of viral proteins among the three samples examined from this animal was different: in the first asinus swab (23_23(MOS)-a.1), coronavirus peptides were absent; in the second asinus swab (23_23(MOS)-a.2), collected the following day, only nucleoprotein peptides were detected; in the fecal sample (23_23(MOS)-F.2), obtained on the second day, membrane protein peptides and spike glycoprotein, but not nucleoprotein, were detected.

8 SUPPLEMENTARY CONTIG SEQUENCES

See the Supplementary File S1 to access the contig sequences described in Supplementary Table S2 and Supplementary Section 5.

FIGURE CAPTIONS

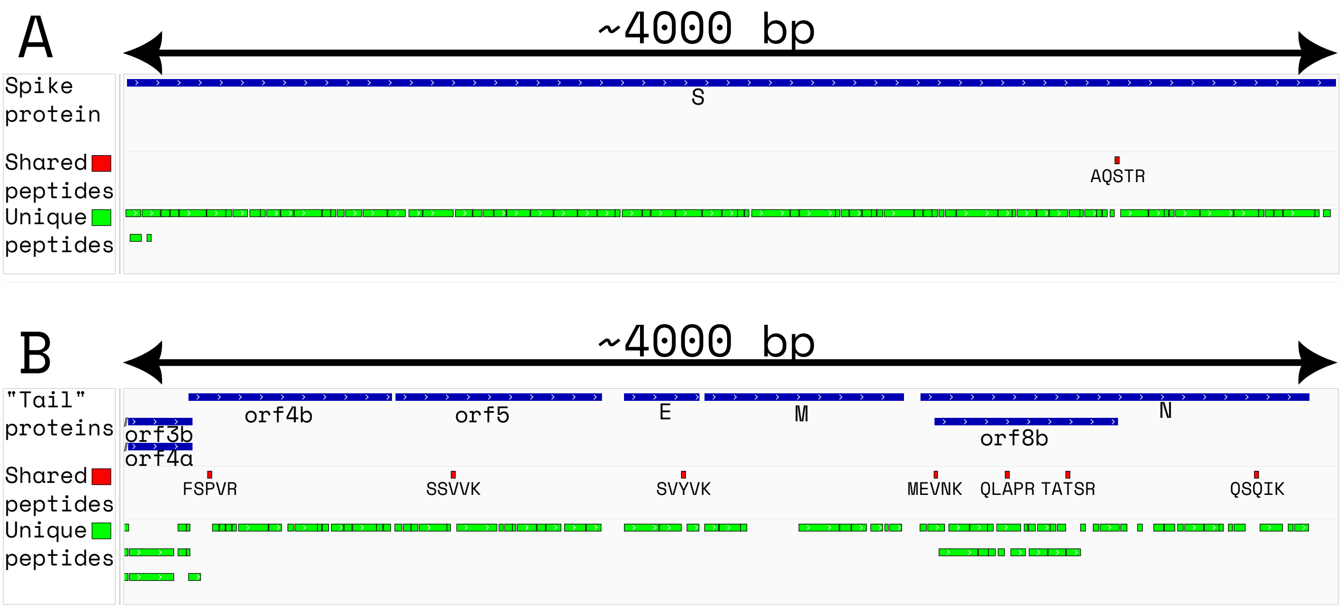

Figure S1. Representation of unique target and shared background peptides on proteins of 23_3(MOS)-a *Coronaviridae* virus. **A**: Spike glycoprotein. **B**: Orf3b and orf4a are shown partially. Represented from IGV using GFF from annotation and peptides' positions written as BED file. Unique peptides are shown in green, shared peptides are shown in red. Sequences of shared peptides are shown in text. Approximate scale lengths (in basepairs) are given above proteins.

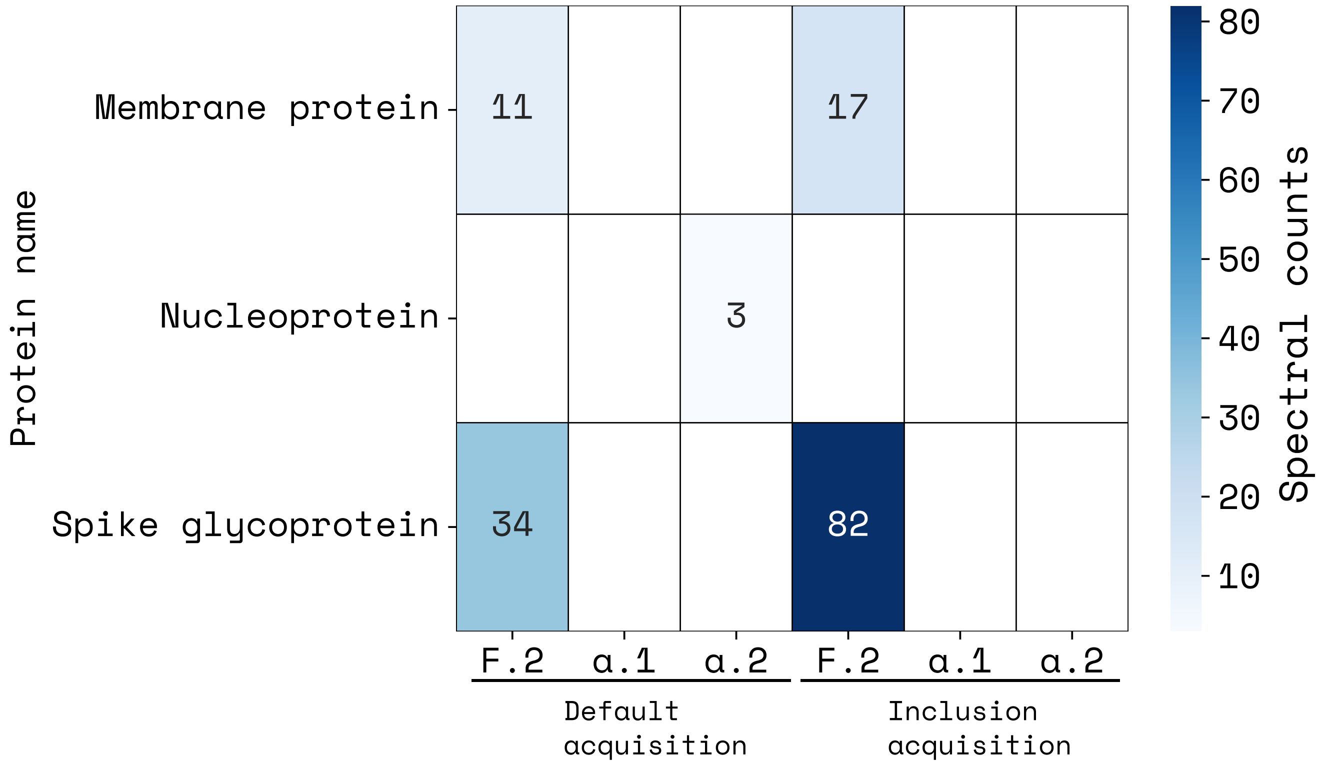

Figure S2. Spectral counts of detected peptides from coronavirus proteins in samples from 23_23(MOS) hedgehog. Cell intensities represent a number of spectral counts. Horizontal labels define the samples from 23_23(MOS), vertical labels define proteins from 23_23(MOS)_F.2.

REFERENCES

Benson, D. A., Cavanaugh, M., Clark, K., Karsch-Mizrachi, I., Lipman, D. J., Ostell, J., et al. (2012). Genbank. *Nucleic acids research* 41, D36–D42

Camacho, C., Coulouris, G., Avagyan, V., Ma, N., Papadopoulos, J., Bealer, K., et al. (2009). Blast+: architecture and applications. *BMC bioinformatics* 10, 421

De Castro, E., Hulo, C., Masson, P., Auchincloss, A., Bridge, A., and Le Mercier, P. (2024). Viralzone 2024 provides higher-resolution images and advanced virus-specific resources. *Nucleic Acids Research* 52, D817–D821

Hodge, K., Ten Have, S., Hutton, L., and Lamond, A. I. (2013). Cleaning up the masses: exclusion lists to reduce contamination with hplc-ms/ms. *Journal of proteomics* 88, 92–103

Jaffe, J. D., Keshishian, H., Chang, B., Addona, T. A., Gillette, M. A., and Carr, S. A. (2008). Accurate inclusion mass screening: a bridge from unbiased discovery to targeted assay development for biomarker verification. *Molecular & Cellular Proteomics* 7, 1952–1962

Langmead, B. and Salzberg, S. L. (2012). Fast gapped-read alignment with bowtie 2. *Nature methods* 9, 357–359

Li, H. (2018). Minimap2: pairwise alignment for nucleotide sequences. *Bioinformatics* 34, 3094–3100

Seemann, T. (2014). Prokka: rapid prokaryotic genome annotation. *Bioinformatics* 30, 2068–2069

Shean, R. C., Makhsous, N., Stoddard, G. D., Lin, M. J., and Greninger, A. L. (2019). Vapid: a lightweight cross-platform viral annotation pipeline and identification tool to facilitate virus genome submissions to ncbi genbank. *BMC bioinformatics* 20, 48

Zell, R., Delwart, E., Gorbalenya, A., Hovi, T., King, A., Knowles, N., et al. (2017). Ictv virus taxonomy profile: Picornaviridae. *Journal of General Virology* 98, 2421–2422
